# Roles of G proteins and their GTPase-activating proteins in platelets

**DOI:** 10.1042/BSR20231420

**Published:** 2024-05-29

**Authors:** Lorna O'Donoghue, Albert Smolenski

**Affiliations:** 1 UCD School of Medicine, University College Dublin, UCD Conway Institute, Belfield, Dublin 4, Ireland; 2 Irish Centre for Vascular Biology, Royal College of Surgeons in Ireland, 123 St. Stephen’s Green 123, Dublin 2, Ireland

**Keywords:** GTPase-activating protein, GTPases, platelet adhesion and activation, signalling

## Abstract

Platelets are small anucleate blood cells supporting vascular function. They circulate in a quiescent state monitoring the vasculature for injuries. Platelets adhere to injury sites and can be rapidly activated to secrete granules and to form platelet/platelet aggregates. These responses are controlled by signalling networks that include G proteins and their regulatory guanine nucleotide exchange factors (GEFs) and GTPase-activating proteins (GAPs). Recent proteomics studies have revealed the complete spectrum of G proteins, GEFs, and GAPs present in platelets. Some of these proteins are specific for platelets and very few have been characterised in detail. GEFs and GAPs play a major role in setting local levels of active GTP-bound G proteins in response to activating and inhibitory signals encountered by platelets. Thus, GEFs and GAPs are highly regulated themselves and appear to integrate G protein regulation with other cellular processes. This review focuses on GAPs of small G proteins of the Arf, Rab, Ras, and Rho families, as well as of heterotrimeric G proteins found in platelets.

## Introduction to platelet signalling

**Figure 1:**
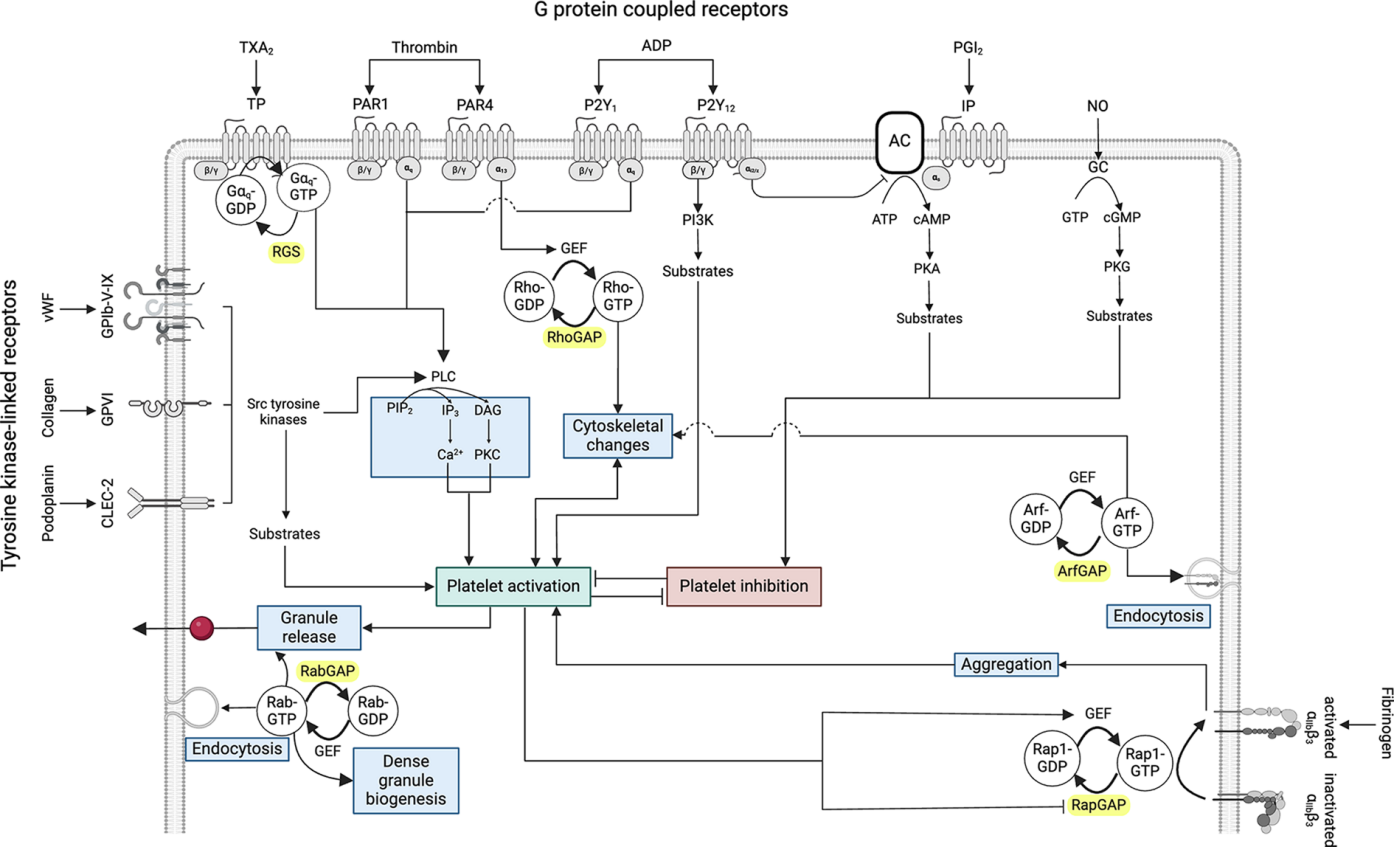
Overview of platelet signalling Platelet can be activated by von Willebrand factor (vWF) binding to the glycoprotein (GP) complex GPIb/V/IX, collagen binding to GPVI, and podoplanin binding to the C-type lectin-like receptor (CLEC-2) which activate tyrosine kinase signalling leading to activation of phospholipase C (PLC), formation of inositol 1,4,5-triphosphate (IP_3_) and 1,2-diacylglycerol (DAG) from phosphatidylinositol 4,5-bisphosphate (PIP_2_), activation of protein kinase C (PKC), and release of Ca^2+^ ions from intracellular stores. Thromboxane A_2_ (TXA_2_), thrombin, and adenosine-diphosphate (ADP) activate TP, protease-activated receptors (PAR1, PAR4 in humans), and purinergic P2Y_1_ and P2Y_12_ G-protein coupled receptors leading to activation of α and βγ subunits of heterotrimeric G proteins by exchange of Gα-bound guanosine-5'-diphosphate, GDP, by guanosine-5'-triphoshate, GTP followed by PLC activation and Ca^2+^ signalling, activation of guanine-nucleotide exchange factors (GEF) of the small G protein RhoA, activation of phosphatidylinositol 3-kinase (PI3K), or inhibition of adenylate cyclase (AC), as indicated, and platelet activation. Gα-GTP is turned into inactive Gα-GDP by regulator of G protein signalling proteins (RGS). RhoA-GTP triggers a range of cytoskeletal rearrangements and RhoA-GTP is turned into inactive RhoA-GDP by GTPase-activation proteins (RhoGAP). Endothelium-derived prostacyclin (PGI_2_) inhibits platelets through activation of the IP receptor coupled to the stimulatory Gα_s_ protein leading to formation of 3'5'-cyclic adenosine monophosphate (cAMP) from adenosine-5'-triphosphate (ATP), activation of protein kinase A (PKA) and phosphorylation of a wide range of substrate proteins. In parallel, endothelial nitric oxide (NO) diffuses through the plasma membrane and activates NO-sensitive guanylate cyclase (GC) to generate 3'5'-cyclic guanosine monophosphate (cGMP) which activates protein kinase G (PKG) to phosphorylate substrates leading to platelet inhibition. Platelet activation and inhbition pathways interact at multiple levels. Platelet activation leads to activation of the Rap1 G protein which enables the transition of inactive to active integrin α_IIb_β_3_ (α_IIb_β_3_) leading to fibrinogen binding, platelet aggregation, and further platelet activation. Rap1 is regulated by specific GEF and GAP proteins. Arf family G proteins are involved in endocytosis of integrin α_IIb_β_3_ and in the regulation of the cytoskeleton, and in other membrane and vesicle transport processes. Rab family G proteins are required for the generation, transport and release of platelet granules, and are probably involved in many other membrane and vesicle transport processes as well as in endocytosis. Arf and Rab proteins are controlled by dedicated GEFs and GAPs. Created with Biorender.com.

Platelets circulate in the bloodstream supporting vascular integrity, haemostasis, immune defence, and tissue repair [1]. Platelets are anucleate and have a lifespan of around 10 days. Core structural components include an open canalicular system which is continuous with the plasma membrane and a dense tubular system involved in Ca^2+^ storage. Platelets contain 50–80 alpha granules, 3–8 dense granules, and 1–3 lysosomal granules, as well as 5–8 mitochondria [2,3]. Each granule type carries specific regulatory molecules which upon exocytosis stimulate a positive feedback loop recruiting more platelets to lesion sites and supporting their activation. Platelet alpha granules contain more than 300 different proteins while dense granules contain ATP, ADP, Ca^2+^ ions, and pyrophosphates which are essential for normal haemostasis [9]. Activated platelets change their shape, they adhere to sites of vascular injury and bind to each other to form aggregates [4]. These processes require rapid and extensive remodelling of the cytoskeleton. Furthermore, activated platelets alter the composition of the outer layer of their plasma membrane by exposing phosphatidylserine thus supporting binding and activation of clotting factors leading to extracellular fibrin fibre formation [5]. Platelet functions are tightly controlled by activating and inhibitory signalling networks (Figure 1) [6].

Platelet activation can be initiated by subendothelial collagen exposed at injury sites providing a binding site for von Willebrand factor (vWF). vWF binding to the glycoprotein (GP) receptor complex GPIb-V-IX establishes platelet tethering but cannot support stable adhesion. Instead, it facilitates platelet binding to collagen via the tyrosine kinase linked GPVI receptor [7]. GPVI is covalently bound to the Fc gamma receptor (FcRγ) and binding of collagen to GPVI induces phosphorylation of an immunoreceptor tyrosine-based activation motif (ITAM) in FcRγ by Src family kinases (Figure 1). This phosphorylation activates the tyrosine kinase Syk leading to hydrolysis of phosphatidylinositol 4,5-bisphosphate (PIP_2_) by phospholipase C γ2 (PLCγ2) resulting in the second messengers 1,2-diacylglycerol (DAG) and inositol 1,4,5-triphosphate (IP_3_). DAG activates protein kinase C (PKC) whereas IP_3_ increases intracellular Ca^2+^ both leading to platelet activation and granule release [8]. ADP, a dense granule component, binds to and activates the G-protein coupled receptors (GPCR) P2Y_12_ and P2Y_1_ leading to amplified platelet activation through reduced cyclic adenosine monophosphate (cAMP) synthesis, phosphatidylinositol 3-kinase (PI3K) activation and through increased Ca^2+^ signalling (Figure 1, see chapter on heterotrimeric G proteins below for further details). Other amplifiers of platelet activation include thromboxane A_2_ (TXA_2_) which activates the thromboxane-prostanoid (TP) GPCR and thrombin, produced in the clotting cascade, which activates platelets through PAR1 and PAR4 GPCRs [9]. Platelet activation leads to a conformational change in the abundant fibrinogen receptor at the plasma membrane, integrin α_IIb_β_3_, which mediates platelet aggregation.

Platelet inhibition is maintained predominantly by the activation of two cyclic nucleotide signalling pathways, one involving cAMP which is activated by endothelium-derived prostacyclin (PGI_2_), and the other cyclic guanosine monophosphate (cGMP) activated by endothelial nitric oxide (NO) (Figure 1). These pathways are likely to be constitutively active due to the continuous production and release of PGI_2_ and NO from the endothelium. PGI_2_ binds to the IP receptor, a GPCR coupled to Gα_s_ which activates adenylate cyclase (AC). AC uses ATP to synthesise cAMP which binds to the regulatory subunit of protein kinase A (PKA) allowing the catalytic subunits to phosphorylate numerous substrate proteins and inhibit platelet activation. NO activates the cGMP pathway following its diffusion through the membrane and subsequent binding to NO-dependent guanylate cyclase which activates protein kinase G (PKG). There is significant overlap between the substrates phosphorylated by PKG and PKA. The inhibitory effects of these pathway are so potent that activation of either pathway can reverse platelet aggregation resulting in dissolution of pre-existing platelet aggregates [10].

Activating and inhibitory pathways converge on guanine-nucleotide binding proteins (G proteins or GTPases) of the Ras superfamily which exert their functions through interactions of the GTP-bound G proteins with downstream effector proteins (Figure 1) [11]. G proteins require guanine nucleotide exchange factors (GEFs) to allow GTP to replace GDP in the active centre. GTPase-activating proteins (GAPs) play a critical role in activating the low endogenous GTPase activities of many G proteins by turning the active GTP-bound forms into their inactive GDP-bound versions. Thus, GAPs effectively serve as off-switches of G protein signalling [12]. This review will provide a broad overview of GAPs of Ras superfamily small G proteins and of alpha subunits of heterotrimeric (αβγ) G proteins found in human platelets. A few GAPs will be described in more detail focusing on available platelet data. Emerging roles of GAPs beyond G protein regulation will be highlighted. Information on platelet GEFs of the Rho family, a member of the Ras superfamily, is included in other reviews [13–16].

## Expression of G proteins and GAPs in platelets

The Ras superfamily of small G proteins encompasses Arf, Rab, Ras, and Rho family proteins and each family has its dedicated GAPs. Similarly, the activity of heterotrimeric (αβγ) G proteins is controlled by regulator of G protein signalling (RGS) proteins. Comprehensive proteomics mapping [17,18] has revealed the G proteins and GAPs expressed by human platelets including protein copy numbers per platelet (Tables 1 and 2). G protein specificities have only been established for a few GAPs and even fewer studies have investigated platelet specific roles of GAPs. GAPs have been identified that regulate Rap1, RhoA, Rac1, and Gαi and Gαq in platelets; however, many orphan G proteins are still present, especially in the Arf and Rab families. Almost all ArfGAPs encoded by the human genome, except ADAPs [19], and most Ras/RapGAPs are expressed in platelets. In contrast, platelets express only about half of the encoded RabGAPs, RhoGAPs, and RGSs. Similar numbers of Arf, Ras and Gα proteins and their GAPs can be found, whereas more Rabs and fewer Rhos compared to their GAPs are expressed. A low Rho/RhoGAP ratio might indicate that individual Rho proteins are targeted by more than one RhoGAP possibly contributing to the creation of diverse zones of local Rho protein activity [20,21]. G protein expression levels vary more than 10-fold with certain G proteins ranging between 10,000 up to above 100,000 copies per platelet. In contrast, GAP copy numbers are generally below 10,000 copies per platelet [18]. A comparison of tissue expression patterns suggests that the G proteins Rab27B, Rab32, Rab37, RhoF, Gα_13_, Gα_q_ (Table 1), and the GAP family members RASA3, Rap1GAP2, RhoGAP6, RhoGAP18, and RGS18 are particularly highly expressed in platelets possibly indicating platelet specific functions (Table 2).

**Table 1:** Overview of G proteins and their expression in human platelets

UniProt	Gene Name	Protein name	Protein copy number per platelet	Platelet specificity	Platelet studies
**Arf family**					
P84077	ARF1	ADP-ribosylation factor 1	49771		
P61204	ARF3	ADP-ribosylation factor 3	44255		
P18085	ARF4	ADP-ribosylation factor 4	33268		
P84085	ARF5	ADP-ribosylation factor 5	36244	+	
P62330	ARF6	ADP-ribosylation factor 6	6370		22916275 26738539
Q13795	ARFRP1	ADP-ribosylation factor-related protein 1	1495		
P40616	ARL1	ADP-ribosylation factor-like protein 1	2274		
P36404	ARL2	ADP-ribosylation factor-like protein 2	922		
P36405	ARL3	ADP-ribosylation factor-like protein 3	3335		
P40617	ARL4A	ADP-ribosylation factor-like protein 4A	-		
P56559	ARL4C	ADP-ribosylation factor-like protein 4C	-		
P49703	ARL4D	ADP-ribosylation factor-like protein 4D	-		
Q9Y689	ARL5A	ADP-ribosylation factor-like protein 5A	Detected		
Q96KC2	ARL5B	ADP-ribosylation factor-like protein 5B	Detected		
A6NH57	ARL5C	Putative ADP-ribosylation factor-like protein 5C	-		
Q9H0F7	ARL6	ADP-ribosylation factor-like protein 6	Detected		
Q96BM9	ARL8A	ADP-ribosylation factor-like protein 8A	4977		
Q9NVJ2	ARL8B	ADP-ribosylation factor-like protein 8B	5894		
Q6T311	ARL9	ADP-ribosylation factor-like protein 9	-		
Q8N8L6	ARL10	ADP-ribosylation factor-like protein 10	-		
Q969Q4	ARL11	ADP-ribosylation factor-like protein 11	-		
Q5H913	ARL13A	ADP-ribosylation factor-like protein 13A	-		
Q3SXY8	ARL13B	ADP-ribosylation factor-like protein 13B	Detected		
Q8N4G2	ARL14	ADP-ribosylation factor-like protein 14	-		
Q9NXU5	ARL15	ADP-ribosylation factor-like protein 15	1838		
Q0P5N6	ARL16	ADP-ribosylation factor-like protein 16	-		
Q8IVW1	ARL17A	ADP-ribosylation factor-like protein 17	-		
**Rab family**					
P62820	RAB1A	Ras-related protein Rab-1A	24879		
Q9H0U4	RAB1B	Ras-related protein Rab-1B	Detected	+	29632235
P61019	RAB2A	Ras-related protein Rab-2A	11553		
Q8WUD1	RAB2B	Ras-related protein Rab-2B	7403		
P20336	RAB3A	Ras-related protein Rab-3A	3735		
P20337	RAB3B	Ras-related protein Rab-3B	-		
Q96E17	RAB3C	Ras-related protein Rab-3C	4173		
O95716	RAB3D	Ras-related protein Rab-3D	Detected		
P20338	RAB4A	Ras-related protein Rab-4A	6013		10938270
P61018	RAB4B	Ras-related protein Rab-4B	6242		
P20339	RAB5A	Ras-related protein Rab-5A	6189		34732055
P61020	RAB5B	Ras-related protein Rab-5B	7661		
P51148	RAB5C	Ras-related protein Rab-5C	12138		
P20340	RAB6A	Ras-related protein Rab-6A	20898		
Q9NRW1	RAB6B	Ras-related protein Rab-6B	27455		
Q9H0N0	RAB6C	Ras-related protein Rab-6C	-		
Q53S08	RAB6D	Ras-related protein Rab-6D	-		
P51149	RAB7A	Ras-related protein Rab-7a	18761		
Q96AH8	RAB7B	Ras-related protein Rab-7b	-		
O14966	RAB29	Ras-related protein Rab-7L1	Detected		
P61006	RAB8A	Ras-related protein Rab-8A	17938	+	23140275
Q92930	RAB8B	Ras-related protein Rab-8B	16055		
P51151	RAB9A	Ras-related protein Rab-9A	1737		
Q9NP90	RAB9B	Ras-related protein Rab-9B	2464		
P61026	RAB10	Ras-related protein Rab-10	23418		
P62491	RAB11A	Ras-related protein Rab-11A	27698		
Q15907	RAB11B	Ras-related protein Rab-11B	25996		
Q6IQ22	RAB12	Ras-related protein Rab-12	2122		
P51153	RAB13	Ras-related protein Rab-13	11203		
P61106	RAB14	Ras-related protein Rab-14	23180		
P59190	RAB15	Ras-related protein Rab-15	8452		
Q9H0T7	RAB17	Ras-related protein Rab-17	-		
Q9NP72	RAB18	Ras-related protein Rab-18	4663		
A4D1S5	RAB19	Ras-related protein Rab-19	-		
Q9NX57	RAB20	Ras-related protein Rab-20	1462		
Q9UL25	RAB21	Ras-related protein Rab-21	5612		
Q9UL26	RAB22A	Ras-related protein Rab-22A	1906		
Q9ULC3	RAB23	Ras-related protein Rab-23	Detected		
Q969Q5	RAB24	Ras-related protein Rab-24	885		
P57735	RAB25	Ras-related protein Rab-25	-		
Q9ULW5	RAB26	Ras-related protein Rab-26	-		
P51159	RAB27A	Ras-related protein Rab-27A	7688		12070017
O00194	RAB27B	Ras-related protein Rab-27B	35939	++	17384153
P51157	RAB28	Ras-related protein Rab-28	Detected		
Q15771	RAB30	Ras-related protein Rab-30	4724	+	
Q13636	RAB31	Ras-related protein Rab-31	2157		35839075
Q13637	RAB32	Ras-related protein Rab-32	8860	++	31399401
Q14088	RAB33A	Ras-related protein Rab-33A	1964		
Q9H082	RAB33B	Ras-related protein Rab-33B	1589		
Q9BZG1	RAB34	Ras-related protein Rab-34	Detected		
Q15286	RAB35	Ras-related protein Rab-35	5614		
O95755	RAB36	Ras-related protein Rab-36	-		
Q96AX2	RAB37	Ras-related protein Rab-37	9153	++	
P57729	RAB38	Ras-related protein Rab-38	4450	+	31399401
Q14964	RAB39A	Ras-related protein Rab-39A	Detected		
Q96DA2	RAB39B	Ras-related protein Rab-39B	Detected		
Q8WXH6	RAB40A	Ras-related protein Rab-40A	-		
Q12829	RAB40B	Ras-related protein Rab-40B	-		
Q96S21	RAB40C	Ras-related protein Rab-40C	-		
P0C0E4	RAB40AL	Ras-related protein Rab-40A-like	-		
Q5JT25	RAB41	Ras-related protein Rab-41	-		
Q8N4Z0	RAB42	Ras-related protein Rab-42	-		
Q86YS6	RAB43	Ras-related protein Rab-43	Detected		
Q7Z6P3	RAB44	Ras-related protein Rab-44	-		
Q5HYI8	RABL3	Rab-like protein 3	1041		
Q3YEC7	RABL6	Rab-like protein 6	1360		
Q8IZ41	RASEF	Ras and EF-hand domain-containing protein	-		
Q9UBK7	RABL2A	Rab-like protein 2A	-		
Q9UNT1	RABL2B	Rab-like protein 2B	Detected		
**Ras family**					
Q9NYS0	NKIRAS1	NF-kappa-B inhibitor-interacting Ras-like protein 1	-		
Q9NYR9	NKIRAS2	NF-kappa-B inhibitor-interacting Ras-like protein 2	Detected		
P11233	RALA	Ras-related protein Ral-A	3363		29437579
P11234	RALB	Ras-related protein Ral-B	6791	+	29437579
P62834	RAP1A	Ras-related protein Rap-1A	124810		30131434
P61224	RAP1B	Ras-related protein Rap-1b	154326	+	30131434
P10114	RAP2A	Ras-related protein Rap-2a	2921		
P61225	RAP2B	Ras-related protein Rap-2b	6248	+	18582561
Q9Y3L5	RAP2C	Ras-related protein Rap-2c	3054		
Q7Z444	ERAS	GTPase ERas	-		
P01112	HRAS	GTPase HRas	Detected		
P01116	KRAS	GTPase KRas	6581		
Q9NYN1	RASL12	Ras-like protein family member 12	-		
O14807	MRAS	Ras-related protein M-Ras	-		
P01111	NRAS	GTPase NRas	6581		
Q9H628	RERGL	Ras-related and estrogen-regulated growth inhibitor-like protein	-		
Q96A58	RERG	Ras-related and estrogen-regulated growth inhibitor	-		
Q92963	RIT1	GTP-binding protein Rit1	1415		
Q99578	RIT2	GTP-binding protein Rit2	-		
P62070	RRAS2	Ras-related protein R-Ras2	Detected		29883056
P10301	RRAS	Ras-related protein R-Ras	2648		
Q92737	RASL10A	Ras-like protein family member 10A	-		
Q96S79	RASL10B	Ras-like protein family member 10B	-		
Q6T310	RASL11A	Ras-like protein family member 11A	-		
Q9BPW5	RASL11B	Ras-like protein family member 11B	-		
**Rho family**					
P60953	CDC42	Cell division control protein 42 homolog	27939		20139097
P63000	RAC1	Ras-related C3 botulinum toxin substrate 1	32870		16195235 18704480
P15153	RAC2	Ras-related C3 botulinum toxin substrate 2	27923		16195235
P60763	RAC3	Ras-related C3 botulinum toxin substrate 3	Detected		
O94844	RHOBTB1	Rho-related BTB domain-containing protein 1	-		
Q9BYZ6	RHOBTB2	Rho-related BTB domain-containing protein 2	-		
P61586	RHOA	Transforming protein RhoA	31315		22045984
P62745	RHOB	Rho-related GTP-binding protein RhoB	Detected		
P08134	RHOC	Rho-related GTP-binding protein RhoC	25384		
O00212	RHOD	Rho-related GTP-binding protein RhoD	-		
Q9HBH0	RHOF	Rho-related GTP-binding protein RhoF	5257	++	23359340
P84095	RHOG	Rho-related GTP-binding protein RhoG	5498		24106270
Q15669	RHOH	Rho-related GTP-binding protein RhoH	-		
Q9H4E5	RHOJ	Rho-related GTP-binding protein RhoJ	-		
P17081	RHOQ	Rho-related GTP-binding protein RhoQ	Detected		
Q7L0Q8	RHOU	Rho-related GTP-binding protein RhoU	-		
Q96L33	RHOV	Rho-related GTP-binding protein RhoV	-		
Q92730	RND1	Rho-related GTP-binding protein Rho6	-		
P52198	RND2	Rho-related GTP-binding protein RhoN	-		
P61587	RND3	Rho-related GTP-binding protein RhoE	-		
**Galpha family**					
P29992	GNA11	Guanine nucleotide-binding protein subunit alpha-11	Detected		
Q03113	GNA12	Guanine nucleotide-binding protein subunit alpha-12	Detected		
Q14344	GNA13	Guanine nucleotide-binding protein subunit alpha-13	6145	++	14528298 15326177
O95837	GNA14	Guanine nucleotide-binding protein subunit alpha-14	-		
P30679	GNA15	Guanine nucleotide-binding protein subunit alpha-15	Detected		
P63096	GNAI1	Guanine nucleotide-binding protein G(i) subunit alpha-1	10226		
P04899	GNAI2	Guanine nucleotide-binding protein G(i) subunit alpha-2	14795		20852125
P08754	GNAI3	Guanine nucleotide-binding protein G(i) subunit alpha-3	8712		
P38405	GNAL	Guanine nucleotide-binding protein G(olf) subunit alpha	-		
P09471	GNAO1	Guanine nucleotide-binding protein G(o) subunit alpha	Detected		
P50148	GNAQ	Guanine nucleotide-binding protein G(q) subunit alpha	14839	++	9296496 15326177
Q5JWF2	GNAS1	Guanine nucleotide-binding protein G(s) subunit alpha isoforms XLas	-		
P63092	GNAS2	Guanine nucleotide-binding protein G(s) subunit alpha isoforms short	2874		18812479 32647264
P11488	GNAT1	Guanine nucleotide-binding protein G(t) subunit alpha-1	Detected		
P19087	GNAT2	Guanine nucleotide-binding protein G(t) subunit alpha-2	-		
A8MTJ3	GNAT3	Guanine nucleotide-binding protein G(t) subunit alpha-3	-		
P19086	GNAZ	Guanine nucleotide-binding protein G(z) subunit alpha	4436	+	

G protein family members were obtained from UniProt and compared with platelet proteome data. Shown are all proteins encoded by the human genome. Protein copy numbers per platelet are given as far as available. ‘Detected’ indicates expressed proteins where a copy number has not yet been determined. All found proteins have also been confirmed at the transcriptome level of megakaryocytes or platelets. ‘-’ indicates that proteins could not be detected in platelets. Platelet specificity was determined as high expression in platelets compared to other human tissues according to https://www.proteomicsdb.org and http://www.humanproteomemap.org (‘+’ indicates within the top 5 highly expressing tissues, ‘++’ indicates platelets as highest expressing tissue in both databases). References for platelet studies on G proteins are given as PubMed ID numbers (PMID).

**Table 2:** Overview of GAPs and their expression in human platelets

UniProt	Gene	Protein name	Protein copy number per platelet	Platelet specificity	Phospho sites	G-protein specificity (cell-based assays)	Functions and regulation (platelet studies)
**ArfGAP, PS50115**							
Q15027	ACAP1	Arf-GAP with coiled-coil, ANK repeat and PH domain-containing protein 1	1150		6	Arf6 (11062263)	
Q15057	ACAP2	Arf-GAP with coiled-coil, ANK repeat and PH domain-containing protein 2	1057		13	Arf6 (11062263)	
Q96P50	ACAP3	Arf-GAP with coiled-coil, ANK repeat and PH domain-containing protein 3	Detected		3		
O75689	ADAP1	Arf-GAP with dual PH domain-containing protein 1	-				
Q9NPF8	ADAP2	Arf-GAP with dual PH domain-containing protein 2	-				
Q9UPQ3	AGAP1	Arf-GAP with GTPase, ANK repeat and PH domain-containing protein 1	Detected		7		
Q99490	AGAP2	Arf-GAP with GTPase, ANK repeat and PH domain-containing protein 2	1199		15		
Q96P47	AGAP3	Arf-GAP with GTPase, ANK repeat and PH domain-containing protein 3	Detected		8		
Q96P64	AGAP4	Arf-GAP with GTPase, ANK repeat and PH domain-containing protein 4	-				
A6NIR3	AGAP5	Arf-GAP with GTPase, ANK repeat and PH domain-containing protein 5	-				
Q5VW22	AGAP6	Arf-GAP with GTPase, ANK repeat and PH domain-containing protein 6	-				
Q5VUJ5	AGAP7	Putative Arf-GAP with GTPase, ANK repeat and PH domain-containing protein 7	-				
Q5VTM2	AGAP9	Arf-GAP with GTPase, ANK repeat and PH domain-containing protein 9	-				
P52594	AGFG1	Arf-GAP domain and FG repeat-containing protein 1	1825		10		
O95081	AGFG2	Arf-GAP domain and FG repeat-containing protein 2	Detected		2		
Q96P48	ARAP1	Arf-GAP with Rho-GAP domain, ANK repeat and PH domain-containing protein 1	3061		17		
Q8WZ64	ARAP2	Arf-GAP with Rho-GAP domain, ANK repeat and PH domain-containing protein 2	-				
Q8WWN8	ARAP3	Arf-GAP with Rho-GAP domain, ANK repeat and PH domain-containing protein 3	-				
Q8N6T3	ARFGAP1	ADP-ribosylation factor GTPase-activating protein 1	Detected		17	Arf1 (19015319, 36513395)	
Q8N6H7	ARFGAP2	ADP-ribosylation factor GTPase-activating protein 2	899		13	Arf1 (19015319, 36513395)	
Q9NP61	ARFGAP3	ADP-ribosylation factor GTPase-activating protein 3	Detected		21	Arf1, Arl1 (19015319, 33715220)	
Q9ULH1	ASAP1	Arf-GAP with SH3 domain, ANK repeat and PH domain-containing protein 1	3588	+	18		Binds Crkl and to focal adhesions (12522101)
O43150	ASAP2	Arf-GAP with SH3 domain, ANK repeat and PH domain-containing protein 2	4132	+	10		
Q8TDY4	ASAP3	Arf-GAP with SH3 domain, ANK repeat and PH domain-containing protein 3	Detected		5		
Q9Y2X7	GIT1	ARF GTPase-activating protein GIT1	3149		30	Arf1, Arf6 (28981399)	Binds RhoGEF6 and RhoGEF7, interacts with integrin αIIbβ3 (26507661, 18211801)
Q14161	GIT2	ARF GTPase-activating protein GIT2	1071		28		
Q8IYB5	SMAP1	Stromal membrane-associated protein 1	890		0		
Q8WU79	SMAP2	Stromal membrane-associated protein 2	1717		5		
**TBC_RabGAP, PS50086**							
Q96CN4	EVI5L	EVI5-like protein	648		2		
O60447	EVI5	Ecotropic viral integration site 5 protein homolog	-				
Q5TC63	GRTP1	Growth hormone-regulated TBC protein 1	Detected		1		
Q5R372	RABGAP1L	Rab GTPase-activating protein 1-like	615		5		
Q9Y3P9	RABGAP1	Rab GTPase-activating protein 1	1619		9		
Q2NKQ1	SGSM1	Small G protein signalling modulator 1	-				
O43147	SGSM2	Small G protein signalling modulator 2	-				
Q96HU1	SGSM3	Small G protein signalling modulator 3	-				
Q86TI0	TBC1D1	TBC1 domain family member 1	Detected		24		
Q9BYX2	TBC1D2	TBC1 domain family member 2A	-				
Q9UPU7	TBC1D2B	TBC1 domain family member 2B	Detected		10		
Q8IZP1	TBC1D3	TBC1 domain family member 3	-				
A6NDS4	TBC1D3B	TBC1 domain family member 3B	-				
Q6IPX1	TBC1D3C	TBC1 domain family member 3C	-				
A0A087WVF3	TBC1D3D	TBC1 domain family member 3D	-				
A0A087X179	TBC1D3E	TBC1 domain family member 3E	-				
A6NER0	TBC1D3F	TBC1 domain family member 3F	-				
Q6DHY5	TBC1D3G	TBC1 domain family member 3G	-				
P0C7X1	TBC1D3H	TBC1 domain family member 3H	-				
A0A087WXS9	TBC1D3I	TBC1 domain family member 3I	-				
A0A087X1G2	TBC1D3K	TBC1 domain family member 3K	-				
B9A6J9	TBC1D3L	TBC1 domain family member 3L	-				
O60343	TBC1D4	TBC1 domain family member 4	Detected		42	Rab10 (33175605)	
Q92609	TBC1D5	TBC1 domain family member 5	1046		27	Rab7B (30111580)	
Q9P0N9	TBC1D7	TBC1 domain family member 7	Detected		2		
O95759	TBC1D8	TBC1 domain family member 8	-				
Q0IIM8	TBC1D8B	TBC1 domain family member 8B	Detected		2		
Q6ZT07	TBC1D9	TBC1 domain family member 9	Detected		0		
Q66K14	TBC1D9B	TBC1 domain family member 9B	1012		18		
Q9BXI6	TBC1D10A	TBC1 domain family member 10A	Detected		14	Rab35 (28566286)	
Q4KMP7	TBC1D10B	TBC1 domain family member 10B	827		22	Rab27b (23671284)	
Q8IV04	TBC1D10C	Carabin	Detected		2		
O60347	TBC1D12	TBC1 domain family member 12	-				
Q9NVG8	TBC1D13	TBC1 domain family member 13	2656	+	1	Rab35 (22762500)	
Q9P2M4	TBC1D14	TBC1 domain family member 14	Detected		5		
Q8TC07	TBC1D15	TBC1 domain family member 15	2534		14		
Q8TBP0	TBC1D16	TBC1 domain family member 16	-				
Q9HA65	TBC1D17	TBC1 domain family member 17	Detected		5	Rab8 (22854040)	
Q8N5T2	TBC1D19	TBC1 domain family member 19	-				
Q96BZ9	TBC1D20	TBC1 domain family member 20	1069		0	Rab1A (22854043)	
Q8IYX1	TBC1D21	TBC1 domain family member 21	-				
Q8WUA7	TBC1D22A	TBC1 domain family member 22A	1138		8		
Q9NU19	TBC1D22B	TBC1 domain family member 22B	Detected		8		
Q9NUY8	TBC1D23	TBC1 domain family member 23	1187		7		
Q3MII6	TBC1D25	TBC1 domain family member 25	Detected		1		
Q86UD7	TBC1D26	TBC1 domain family member 26	-				
Q9UFV1	TBC1D29P	Putative TBC1 domain family member 29	-				
Q9Y2I9	TBC1D30	TBC1 domain family member 30	-				
Q96DN5	TBC1D31	TBC1 domain family member 31	-				
Q8TEA7	TBCK	TBC domain-containing protein kinase-like protein	Detected		1		
P35125	USP6	Ubiquitin carboxyl-terminal hydrolase 6	-				
Q92738	USP6NL	USP6 N-terminal-like protein	Detected		26		
**RasGAP, PS50018**							
Q5VWQ8	DAB2IP	Disabled homolog 2-interacting protein	-				
Q14C86	GAPVD1	GTPase-activating protein and VPS9 domain-containing protein 1	1283		34		
P46940	IQGAP1	Ras GTPase-activating-like protein IQGAP1	1040		17	No GAP activity, Cdc42/Rac1 effector (34830479)	Inhibits alpha granule release (15026422)
Q13576	IQGAP2	Ras GTPase-activating-like protein IQGAP2	8102	+	19	No GAP activity, Cdc42/Rac1 effector (34830479)	Interacts with arp3 and actin (12515716)
Q86VI3	IQGAP3	Ras GTPase-activating-like protein IQGAP3	-				
P21359	NF1	Neurofibromin	628		20	HRas (9668168)	
O95294	RASAL1	RasGAP-activating-like protein 1	-				
Q9UJF2	RASAL2	Ras GTPase-activating protein nGAP	Detected		26		
Q86YV0	RASAL3	RAS protein activator like-3	573		25		
C9J798	RASA4B	Ras GTPase-activating protein 4B	-				
P20936	RASA1	Ras GTPase-activating protein 1	2921	+	5		
Q15283	RASA2	Ras GTPase-activating protein 2	Detected		5		
Q14644	RASA3	Ras GTPase-activating protein 3	8293	++	12	Rap1, H-Ras (16431904)	Reduces Rap1-GTP, inhibits platelet adhesion and aggregation, binds PIP3, linked to P2Y12 receptor via PI3K (24967784, 25705885, 27903653)
O43374	RASA4	Ras GTPase-activating protein 4	-				
Q96PV0	SYNGAP1	Ras/Rap GTPase-activating protein SynGA	-				
**RapGAP, PS50085**							
Q5VVW2	GARNL3	GTPase-activating Rap/Ran-GAP domain-like protein 3	-				
Q6GYQ0	RALGAPA1	Ral GTPase-activating protein subunit alpha-1	669		28		
Q2PPJ7	RALGAPA2	Ral GTPase-activating protein subunit alpha-2	Detected		12		
Q86X10	RALGAPB	Ral GTPase-activating protein subunit beta	884		7	RalA (29915037)	
P47736	RAP1GAP	Rap1 GTPase-activating protein 1	-				
Q684P5	RAP1GAP2	Rap1 GTPase-activating protein 2	2327	++	22	Rap1 (15632203)	Inhibited by S9 phosphorylation, activated by PKA/PKG mediated S7 phosphorylation, binds 14-3-3, binds Slp1 and stimulates dense granule release (15632203, 18039662, 19528539)
O43166	SIPA1L1	Signal-induced proliferation-associated 1-like protein 1	Detected		65		
Q9P2F8	SIPA1L2	Signal-induced proliferation-associated 1-like protein 2	-				
O60292	SIPA1L3	Signal-induced proliferation-associated 1-like protein 3	-				
Q96FS4	SIPA1	Signal-induced proliferation-associated protein 1	Detected		12		
P49815	TSC2	Tuberin	593		35		
**RhoGAP, PS50238**							
Q9Y3L3	SH3BP1	SH3 domain-binding protein 1	595		14		
Q12979	ABR	Active breakpoint cluster region-related protein	Detected		5	Rac1 (32203420)	
Q96P48	ARAP1	Arf-GAP with Rho-GAP domain, ANK repeat and PH domain-containing protein 1	3061		17	Rac1, Cdc42 (32203420)	
Q8WZ64	ARAP2	Arf-GAP with Rho-GAP domain, ANK repeat and PH domain-containing protein 2	-				
Q8WWN8	ARAP3	Arf-GAP with Rho-GAP domain, ANK repeat and PH domain-containing protein 3	-				
P11274	BCR	Breakpoint cluster region protein	793		47	Rac1 (32203420)	
Q6ZT62	BARGIN	Bargin	-				
P15882	CHN1	N-chimaerin	-				
P52757	CHN2	Beta-chimaerin	-				
Q8WUY9	DEPDC1B	DEP domain-containing protein 1B	-				
O94988	FAM13A	Protein FAM13A	-				
Q9NYF5	FAM13B	Protein FAM13B	-				
Q9P107	GMIP	GEM-interacting protein	1228		17	RhoA, Rac1, Cdc42 (32203420)	
P32019	INPP5B	Type II inositol 1,4,5-trisphosphate 5-phosphatase	1629		1		
B2RTY4	MYO9A	Unconventional myosin-IXa	Detected		14		
Q13459	MYO9B	Unconventional myosin-IXb	1428		40	RhoA (33268376)	Activated by PKA/PKG mediated S1354 phosphorylation (32692911)
Q01968	OCRL	Inositol polyphosphate 5-phosphatase OCRL	850		3	RhoA (33528045)	Reduces RhoA-GTP, augments Rac1-GTP and myosin light chain phosphorylation, inhibits filopodia, stimulates spreading, clot retraction and thrombus formation (33528045, 36176266)
O60890	OPHN1	Oligophrenin-1	1527		3	RhoA, Rac1, Cdc42 (25556321)	Reduces RhoA-GTP, Rac1-GTP, and Cdc42-GTP, inhibits adhesion and granule release (25556321)
P27986	PIK3R1	Phosphatidylinositol 3-kinase regulatory subunit alpha	1904		17		
O00459	PIK3R2	Phosphatidylinositol 3-kinase regulatory subunit beta	Detected		12		
Q15311	RALBP1	RalA-binding protein 1	785		14	Rac1 (32203420)	
Q9H0H5	RACGAP1	Rac GTPase-activating protein 1	-				
Q07960	ARHGAP1	Rho GTPase-activating protein 1	9290	+	8	RhoA, Cdc42 (32203420)	
P98171	ARHGAP4	Rho GTPase-activating protein 4	1400		6	Rac1 (32203420)	
Q13017	ARHGAP5	Rho GTPase-activating protein 5	Detected		16	RhoA (32203420)	
O43182	ARHGAP6	Rho GTPase-activating protein 6	4151	++	9	RhoA (32203420)	Binds COPD and COPI vesicles, might regulate protein transport (37409526)
Q96QB1	DLC1	Rho GTPase-activating protein 7	-				
P85298	ARHGAP8	Rho GTPase-activating protein 8	-				
Q9BRR9	ARHGAP9	Rho GTPase-activating protein 9	Detected		3	Rac1 (32203420)	
A1A4S6	ARHGAP10	Rho GTPase-activating protein 10	1114		1	RhoA (32203420)	
Q6P4F7	ARHGAP11A	Rho GTPase-activating protein 11A	-				
Q3KRB8	ARHGAP11B	Inactive Rho GTPase-activating protein 11B	-				
Q8IWW6	ARHGAP12	Rho GTPase-activating protein 12	Detected		23	Rac1 (32203420)	
Q53QZ3	ARHGAP15	Rho GTPase-activating protein 15	833		11	Rac1 (32203420, 32839212)	
Q68EM7	ARHGAP17	Rho GTPase-activating protein 17	1603		21	Rac1, RhoA (22975681)	Regulated by tyrosine phosphorylation (22975681), activated by PKA/PKG mediated S702 phosphorylation (26507661)
Q8N392	ARHGAP18	Rho GTPase-activating protein 18	7100	++	8	RhoC (25425145), RhoA (32016689)	
Q14CB8	ARHGAP19	Rho GTPase-activating protein 19	-				
Q9P2F6	ARHGAP20	Rho GTPase-activating protein 20	-				
Q5T5U3	ARHGAP21	Rho GTPase-activating protein 21	886		46	RhoA (32203420, 33727037), Cdc42 (33727037)	Inhibits alpha granule release and aggregation (33727037)
							
Q7Z5H3	ARHGAP22	Rho GTPase-activating protein 22	-				
Q9P227	ARHGAP23	Rho GTPase-activating protein 23	-				
Q8N264	ARHGAP24	Rho GTPase-activating protein 24	-				
P42331	ARHGAP25	Rho GTPase-activating protein 25	1054		11	Rac1 (36190314)	
Q9UNA1	ARHGAP26	Rho GTPase-activating protein 26	Detected		1	RhoA (32203420)	
Q6ZUM4	ARHGAP27	Rho GTPase-activating protein 27	Detected		11	Rac1 (32203420)	
Q9P2N2	ARHGAP28	Rho GTPase-activating protein 28	-				
Q52LW3	ARHGAP29	Rho GTPase-activating protein 29	-				
Q7Z6I6	ARHGAP30	Rho GTPase-activating protein 30	Detected		15	RhoA, Rac1, Cdc42 (32203420)	
Q2M1Z3	ARHGAP31	Rho GTPase-activating protein 31	-				
A7KAX9	ARHGAP32	Rho GTPase-activating protein 32	Detected		40		
O14559	ARHGAP33	Rho GTPase-activating protein 33	-				
Q9NRY4	ARHGAP35	Rho GTPase-activating protein 35	783		23	RhoA, Rac1 (32203420)	
Q6ZRI8	ARHGAP36	Rho GTPase-activating protein 36	-				
Q9C0H5	ARHGAP39	Rho GTPase-activating protein 39	-				
Q5TG30	ARHGAP40	Rho GTPase-activating protein 40	-				
A6NI28	ARHGAP42	Rho GTPase-activating protein 42	-				
Q17R89	ARHGAP44	Rho GTPase-activating protein 44	-				
Q92619	ARHGAP45	Rho GTPase-activating protein 45 (HMHA1)	3891		25		
Q7Z6B7	SRGAP1	SLIT-ROBO Rho GTPase-activating protein 1	Detected		9	Rac1 (32203420)	
O75044	SRGAP2	SLIT-ROBO Rho GTPase-activating protein 2	Detected		23	Rac1, Cdc42 (32203420, 31880824)	
O43295	SRGAP3	SLIT-ROBO Rho GTPase-activating protein 3	-				
Q9Y3M8	STARD13	StAR-related lipid transfer protein 13	Detected		8	RhoA (32203420)	
Q92502	STARD8	StAR-related lipid transfer protein 8	Detected		3	RhoA, Cdc42 (32203420, 25673874)	
Q6ZW31	SYDE1	Rho GTPase-activating protein SYDE1	-				
Q5VT97	SYDE2	Rho GTPase-activating protein SYDE2	-				
Q8N103	TAGAP	T-cell activation Rho GTPase-activating protein	-				
**RGS, PS50132**							
Q08116	RGS1	Regulator of G-protein signalling 1	-				
P41220	RGS2	Regulator of G-protein signalling 2	-				
P49796	RGS3	Regulator of G-protein signalling 3	Detected		8	Gαi, Gαo (33007266)	
P49798	RGS4	Regulator of G-protein signalling 4	-				
O15539	RGS5	Regulator of G-protein signalling 5	-				
P49758	RGS6	Regulator of G-protein signalling 6	2434	+	0	Gαo (32513692, 33007266)	
P49802	RGS7	Regulator of G-protein signalling 7	-				
P57771	RGS8	Regulator of G-protein signalling 8	-				
O75916	RGS9	Regulator of G-protein signalling 9	-				
O43665	RGS10	Regulator of G-protein signalling 10	4608	+	5	Gαq, Gαi, Gαo (30150297, 33007266)	Inhibits Gαq and Gαi signalling, aggregation, granule release, regulated by spinophilin and 14-3-3 (27829061, 30150297)
O94810	RGS11	Regulator of G-protein signalling 11	-				
O14924	RGS12	Regulator of G-protein signalling 12	-				
O14921	RGS13	Regulator of G-protein signalling 13	-				
O43566	RGS14	Regulator of G-protein signalling 14	Detected		12	Gαi1 (30093406, 33007266)	
O15492	RGS16	Regulator of G-protein signalling 16	-				
Q9UGC6	RGS17	Regulator of G-protein signalling 17	-				
Q9NS28	RGS18	Regulator of G-protein signalling 18	4463	++	3	Gαi, Gαq (33007266)	Inhibits Gαi and Gαq signalling, regulated by spinophilin and 14-3-3, inhibited by S49/S218 phosphorylation, activated by PKA/PKG mediated S216 phosphorylation (22210881, 26407691, 22234696, 24244663)
P49795	RGS19	Regulator of G-protein signalling 19	1086		5	Gαz (33007266)	
O76081	RGS20	Regulator of G-protein signalling 20	-				
Q2M5E4	RGS21	Regulator of G-protein signalling 21	-				
Q8NE09	RGS22	Regulator of G-protein signalling 22	-				
Q15835	GRK1	Rhodopsin kinase GRK1	-				
P25098	GRK2	Beta-adrenergic receptor kinase 1	1409		7		
P35626	GRK3	Beta-adrenergic receptor kinase 2	Detected		3		
P32298	GRK4	G protein-coupled receptor kinase 4	-				
P34947	GRK5	G protein-coupled receptor kinase 5	1557		3	Inhibits PAR1 receptor signalling (34581777)	
P43250	GRK6	G protein-coupled receptor kinase 6	2005		2	Inhibits PAR1 and P2Y12 receptor signalling (31899801)	
Q8WTQ7	GRK7	Rhodopsin kinase GRK7	-				
O15085	ARHGEF11	Rho guanine nucleotide exchange factor 11	-				
O15169	AXIN1	Axin-1	Detected		8		
Q9Y2T1	AXIN2	Axin-2	-				
Q9Y5W8	SNX13	Sorting nexin-13	Detected		0		
Q9Y5W7	SNX14	Sorting nexin-14	Detected		1		
Q9H3E2	SNX25	Sorting nexin-25	-				
O43572	AKAP10	A-kinase anchor protein 10, mitochondrial	819		6	Rab4, Rab11 (19797056)	

Proteins containing GAP domains were obtained from UniProt and compared with platelet proteome data. Shown are all proteins encoded by the human genome. Protein copy numbers per platelet are given as far as available. ‘Detected’ indicates expressed proteins where a copy number has not yet been determined. All found proteins have also been confirmed at the transcriptome level of megakaryocytes or platelets. ‘-’ indicates that proteins could not be detected in platelets. Platelet specificity was determined as high expression in platelets compared to other human tissues according to https://www.proteomicsdb.org and http://www.humanproteomemap.org (‘+’ indicates within the top 5 highly expressing tissues, ‘++’ indicates platelets as highest expressing tissue in both databases). Phosphorylation (Phospho) sites refers to sites identified by proteomics and by low throughput studies. Shown are numbers of phosphorylation sites found in any cell type with a minimum of at least 5 references according to PhosphoSite (https://www.phosphosite.org/). References for G protein specificities of GAPs are given as PubMed ID numbers (PMID). Detailed studies refers to platelet data, listed are PMIDs.

## GAP domain structure and function

**Figure 2:**
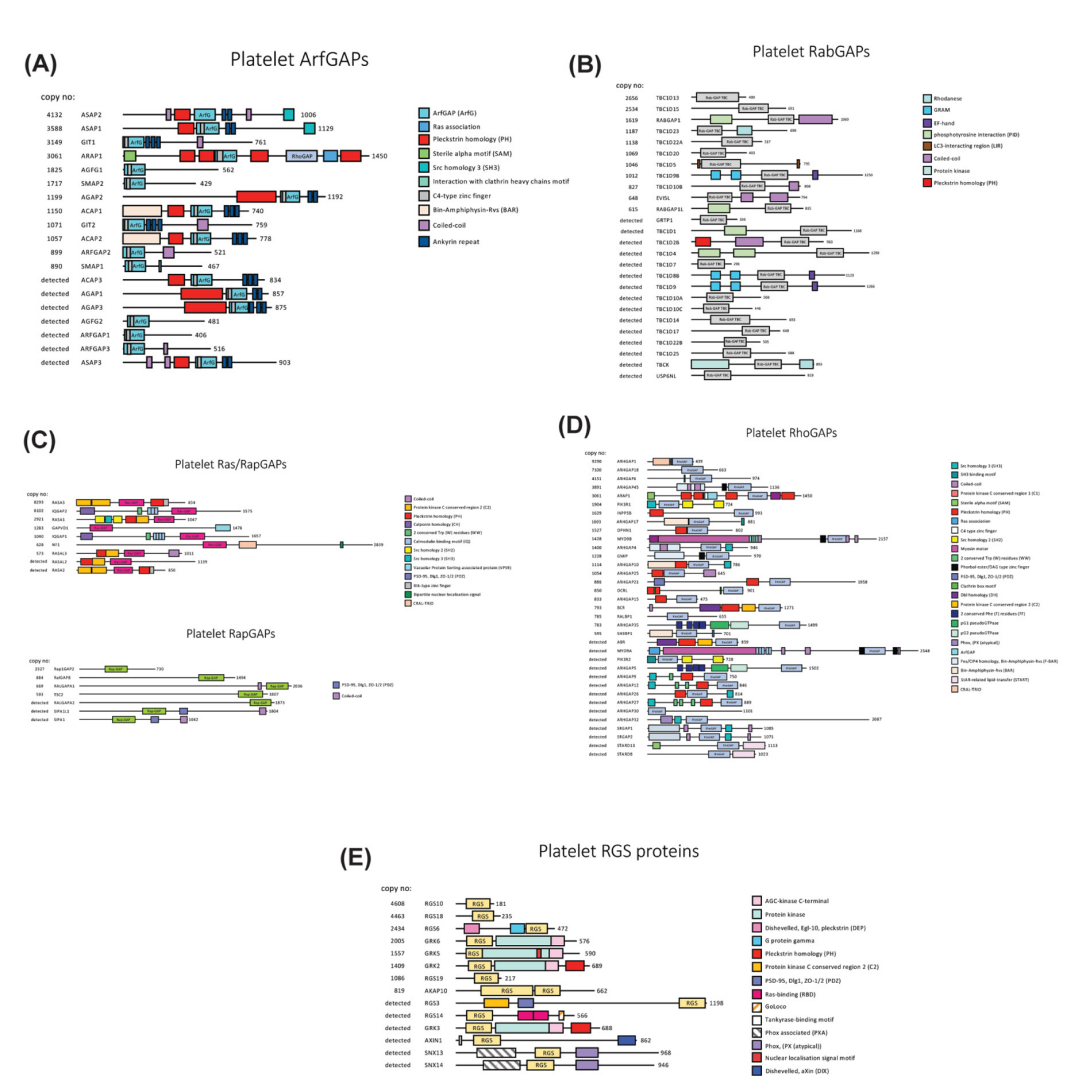
Domain structures of platelet GAPs Shown are GAPs of Arf (**A**), Rab (**B**), Ras/Rap (**C**), Rho (**D**), and alpha subunits of heterotrimeric (**E**) G proteins found in human platelets. Domains, domain boundaries, and protein lenghts are given according to UniProt, and proteins are sorted according to their expression levels (Table 2). Domain names and abbreviations are given.

GAPs are defined as proteins that contain catalytic GAP domains and GAP domain profiles have been defined and are accessible through the Prosite database (ARFGAP, PS50115; TBC_RABGAP, PS50086; RAS_GTPASE_ACTIV_2, PS50018; RAPGAP, PS50085; RHOGAP, PS50238; RGS, PS50132). In general, GAPs bound to their small G proteins contribute catalytic site residues to stimulate GTP hydrolysis. For example, GAPs for Ras and Rho GTPases contribute an essential arginine, called arginine-finger, whereas RabGAPs use an arginine/glutamine dual-finger, and GAPs for Rap and Ral use an asparagine, called asparagine-thumb [12,22,23]. RGS domains do not appear to contribute catalytic site residues but stabilize a pre-transition state of Gα [24,25]. Although minimal sequences exhibiting GAP activity have been defined, in some cases other regions or domains outside of the core GAP domain contribute supporting or inhibitory functions. For example, autoinhibition is a common feature of RhoGAPs [26] which has been confirmed for the platelet GAPs RhoGAP6 [27] and RASA3 [28]. The pleckstrin homology (PH) domain of the ArfGAP ASAP1 supports Arf binding whereas its predicted N-terminal Bin/amphiphysin/Rvs (BAR) domain inhibits GAP activity (Figure 2A) [29]. In contrast, nonmuscle myosin 2A (NM2A), a BAR domain binding partner, stimulates ASAP1 activity, possibly by relieving the autoinhibition [30]. Other examples for regulatory components within GAPs include the RhoA regulator RhoGAP35 which exhibits a protrusion localization sequence, including two pseudo GTPase regions (Figure 2D), involved in localization to lamellipodia in cancer cells that inhibits GAP activity [31].

## Other domains contained in GAPs

**Figure 3:**
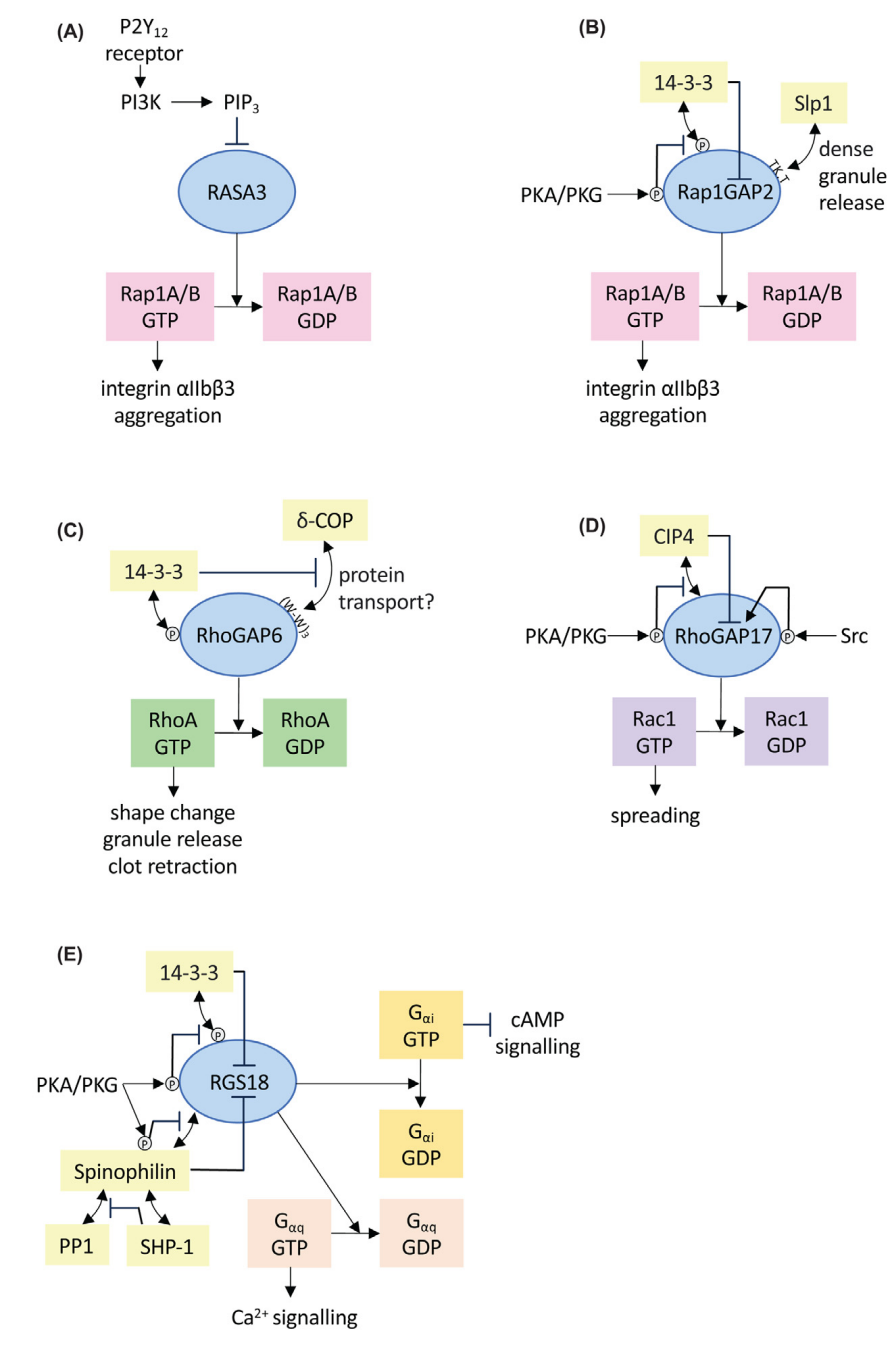
Examples of regulation and function of platelet GAPs (**A**) RASA3 is a Rap1GAP which is inhibited by binding to phosphatidylinositol 3,4,5-tris-phosphate (PIP_3_) at the plasma membrane. PIP3 is generated by phosphatidylinositol 3-kinase (PI3K) downstream of the P2Y_12_ receptor for ADP. In this way ADP could lead to inhibition of RASA3 leading to elevated Rap1-GTP levels stimulating integrin activation and aggregation. (**B**) Rap1GAP2 is inhibited by binding of the adapter protein 14-3-3 to phosphorylated serine 9 of Rap1GAP2. cAMP- and cGMP-dependent protein kinase (PKA, PKG) mediated phosphorylation of serine 7 of Rap1GAP2 inhibits 14-3-3 binding resulting in increased GAP activity, formation of inactive Rap1-GDP and inhibition of aggregation. Rap1GAP2 also interacts with synaptotagmin-like protein 1 (Slp1) through a TKxT motif in the C-terminal part of Rap1GAP2 (T524-K525-X-T527). Slp1 inhibits dense granule release whereas binding of Rap1GAP2 to Slp1 reverses this inhibition. (**C**) The Rho-specific GAP RhoGAP6 interacts with δ-COP, a core component of the COPI vesicle coat, through a triple tryptophane motif ((W-W)_3_). Through this interaction GAP6 is able to regulate intracellular protein transport along the secretory pathway. The exact role of COPI in platelets is not known. At the same time RhoGAP6 is regulated by 14-3-3 binding to serine 37 which has a negative impact on the δ-COP interaction. (**D**) The Rac1-specific GAP RhoGAP17 is regulated by PKA and PKG mediated phosphorylation of serine 702 which interferes with binding to the actin-regulating Cdc42-interacting protein 4 (CIP4). CIP4 is also an effector of Rac1-GTP. Serine 702 phosphorylation stimulates the GAP function of RhoGAP17 presumably by interfering with an inhibitory function of CIP4. In addition, Src family kinases (SFK) can phosphorylate RhoGAP17 on tyrosines 124 and 314 leading to GAP activation. (**E**) RGS18 has GAP activity towards Gα_q_ and Gα_i_. RGS18 is negatively regulated by 14-3-3 and spinophilin. During platelet inhibition PKA and PKG mediated phosphorylation of serine 216 on RGS18 triggers the dephosphorylation of the 14-3-3 binding phospho-serine 218 on RGS18 resulting in detachment of 14-3-3 and activation of RGS18 function. PKA mediated phosphorylation of serine 94 on spinophilin contributes to dissolution of the RGS18/14-3-3/spinophilin complex. Spinophilin also interacts with the serine/threonine phosphatase PP1 and the tyrosine phosphatase SHP-1. PP1 binding to spinophilin is negatively regulated by the SHP-1. During platelet activation SHP-1 detaches from spinophilin enabling PP1 recruitment, de-phosphorylation of PKA/PKG-dependent regulatory sites, enhanced 14-3-3 and spinophilin binding and reduced RGS activity. P refers to protein phosphorylations detected in platelets. Double arrowhead lines refer to protein/protein interactions.

GAPs are typically large proteins including multiple domains (Figure 2). Only few GAPs appear to consist of a catalytic GAP domain alone. These catalytic domain only GAPs include the most highly expressed platelet RabGAP TBC1D13 as well as RGSs 10, 18, and 19 (Figure 2B, 2E). All other RabGAPs and RGSs as well as ArfGAPs, Ras/RapGAPs, and RhoGAPs (Figure 2A, 2C, 2D) contain a diverse set of additional domains. A prominent feature found in most GAPs are domains that can mediate lipid and membrane binding, as for example, PH (most ArfGAPs), C2 (Ras/RapGAPs) and BAR domains (RhoGAP). Membrane binding might be required for the interaction with G proteins as most G proteins are attached to membranes via lipid anchors [11]. Furthermore, a variety of protein/protein interaction domains are present in these GAPs including ankyrin repeats (ArfGAPs), Src homology 2 (SH2), Src homology 3 (SH3), WW, and post synaptic density protein, Drosophila disc large tumor suppressor, and zonula occludens-1 protein (PDZ) domains (Figure 2). Protein interaction domains might localise GAPs to specific G proteins, to other GAPs and GEFs, or to adapter proteins and thereby contribute to the formation of G protein networks required for the coordination of multiple platelet functions [21,32]. Another characteristic seen in platelet GAPs is their ability to regulate other cellular processes independent of their GAP activity. For example sequences outside the catalytic GAP domain enable interactions of Rap1GAP2 with Slp1 to modulate dense granule release (see below for details, Figure 3B) [33]. RhoGAP6 enhances protein transport through the secretory pathway and its RhoGAP activity actually inhibits this effect (Figure 3C) [27].

Most GAPs contain sites for possible post-translational modifications. For example, all platelets GAPs are predicted to contain 10 phosphorylation sites per protein on average (Table 2), estimate based on a conservative approach with a minimum of 5 references according to PhosphoSite) pointing towards complex regulation by multi-site phosphorylation [34]. GAP phosphorylation has been linked to changes in GAP activity during platelet activation and inhibition. Many G proteins are turned into their active GTP-bound versions during platelet activation. Maintenance of GTP-bound G proteins requires reduced GAP activities whereas classical platelet inhibitors tend to stimulate GAPs. This has been observed for RASA3, Rap1GAP2, RhoGAP17, Myo9B, RGS10 and RGS18 (see below for details). These activity changes have been linked to phosphorylation and dephosphorylation of regulatory sites involving binding of 14-3-3 adapter proteins. The adapter protein 14-3-3 is common phosphorylation dependent interaction partner of many GAPs including Rap1GAP2, RhoGAP6, RGS10 and RGS18 (Figure 3) and 14-3-3s play an established role in regulating platelet functions [35].

## Arf proteins and ArfGAPs in platelets

### Arf proteins

G proteins of the Arf family are generally known has signalling molecules controlling diverse cellular functions including vesicle formation and membrane trafficking as well as remodelling of the actin cytoskeleton and cell adhesion [36]. Arf1, 3, 4, and 5 are expressed in high amounts in platelets [18] (Table 1) and are thought to have overlapping roles in vesicle formation and transport to and from the Golgi and in recycling endosomes [37–39]. For example, Arf1 initiates vesicle formation by recruiting coat protein complex I (COPI) and clathrin adapter proteins (AP-1, AP-3, and AP-4) to membranes. Coat proteins then complete vesicle formation and select protein and lipid cargo for vesicular transport [37]. Of all 27 Arf proteins found in platelets only Arf6 has been studied is some detail. Arf6 has a role in controlling signalling in the cell periphery. In platelets, Arf6 is involved in endocytosis of membrane receptors including the fibrinogen receptor integrin α_IIb_β_3_, the P2Y_12_ ADP receptor [40] and Toll-like receptors 7 and 9 [41]. Reduced Arf6 expression in mouse platelets resulted in impaired fibrinogen endocytosis and uptake into alpha granules which correlated with increased platelet spreading and clot retraction [42]. In contrast with many other small G proteins Arf6 is typically found in its GTP-bound form in resting platelets. Arf6 transitions to the inactive GDP-bound form during platelet activation by collagen and thrombin [43], although one study has also described the opposite effect [44]. The ArfGAP regulating Arf6 in platelets is unknown. *In vitro* studies using purified proteins have provided evidence for specificity of GIT1, ACAP1, and ACAP2 towards Arf6 [45,46], and these three GAPs are present in platelets (Figure 2A). High Arf6-GTP levels might be maintained by cytohesin-2 which has been identified as GEF for Arf6 in platelets [47].

### ArfGAPs

Interestingly, the most highly expressed ArfGAPs in platelets, ASAP1, ASAP2, and GIT1 (Figure 2A) have been described as integrin and receptor regulators [36,48]. Integrin regulation is thought to be mediated by effects on membrane trafficking and endocytosis possibly via Arf6 or other Arfs, or through Rac1 signalling. ASAP1 localizes to platelet focal adhesions through binding of the adapter protein CrkL [49]. GIT1 currently is the most extensively studied ArfGAP in platelets. Platelet activation has been shown to induce tyrosine phosphorylation of GIT1 probably through integrin α_IIb_β_3_ induced outside-in signalling mediated by Src kinase [50]. Tyrosine phosphorylation correlates with translocation of GIT from a membrane to a cytoskeletal fraction together with integrin β3. As GIT1 is known to bind constitutively to the Rac1 regulators RhoGEF6 [51] and RhoGEF7 [50] in platelets cytoskeletal recruitment could contribute to Rac1 and possibly Arf6 mediated actin rearrangements during platelet activation. ARAP1, another highly expressed platelet ArfGAP (Figure 2A), contains a RhoGAP domain as well as a Ras-association region and might support the coordination of the function of multiple G proteins including Rap1, Rac1, RhoA, Cdc42, and Arf proteins [52]. Similar to other ArfGAPs ARAP1 contains PH domains (Figure 2A) which might facilitate close association to membrane surfaces required for effective Arf interaction [53].

In summary, platelet Arfs are only marginally described apart from Arf6 which has a role in receptor endocytosis and in the regulation of the actin cytoskeleton. As ArfGAPs appear to be recruiting additional G protein regulators future studies should consider analysing Arfs in combination with their GAPs (and GEFs) to improve understanding of the specific roles of Arf proteins in platelet signalling [36].

## Rab proteins and RabGAPs in platelets

### Rab proteins

Similar to Arfs Rab proteins are involved in controlling intracellular membrane and vesicle traffic. Rabs tend to associate with specific vesicles thus conferring membrane identity and enabling directed transport between donor and acceptor compartments, both in secretion as well as in endocytosis and recycling pathways [54,55]. For example, active Rab27-GTP localised at the cytosolic surface of platelet dense granules can bind to the effector protein Munc13-4 supporting tethering of the granule to the plasma membrane [56]. Tethering is followed by soluble N-ethylmaleimide-sensitive factor attachment protein receptor (SNARE) mediated membrane fusion and secretion of granule content [57]. Prominent vesicle/granule associated SNAREs in platelets are VAMP-8 and VAMP-7 which interact with target membrane SNAREs like syntaxin-11 and SNAP-23 as well as Munc18 proteins. The Ras family G protein Ral might be another component of the secretory complex through interactions with SNARE binding exocyst factors. However, mouse knockout studies suggest only minor roles for RalA and RalB in granule release, but a possible role for Ral in P-selectin translocation to the plasma membrane has been proposed [58]. Rab27B is the most highly expressed Rab in platelets [18] (Table 1) and its role in dense granule release has been confirmed [59]. Similar to Arf6 Rab27 is present predominantly in the GTP-bound form in unstimulated platelets, whereas granule release leads to a decrease of Rab27-GTP levels [60]. A possible GAP that could mediate Rab27 inactivation is TBC1D10B which has been shown to have activity towards Rab27B in other cells [61]. Rab8 might also support tethering of dense granules to the plasma membrane by interacting with synaptotagmin-like protein 4 [62]. Regarding the role of Rab proteins in the biogenesis of dense granules data have been obtained from Hermansky–Pudlak syndrome (HPS) patients and mouse models. Combined deficiency of Rab32 and Rab38 in mice leads to defective dense granule formation and impaired platelet function similar to defects seen in HPS patients [63]. Alpha granules were not affected by Rab32/38 deficiency and platelet counts were not changed indicating that dense granule deficiency does not necessarily impact on platelet biogenesis. These data support earlier findings obtained using the megakaryocytic cell line MEG-01 suggesting that Rab32 and Rab38 enable the transport of dense granule components from early endosomes via AP-3 and Rab7 labelled vesicles towards dense granule precursors [64]. Little data are available regarding biogenesis, tethering and release of alpha granules. Rab1B is down-regulated in patients with alpha granule defects due to deficiency of the transcription factor RUNX1 and Rab1B was shown to be involved in ER-Golgi transport of alpha granule content in megakaryocytic cells; however, changes in alpha granule release were not reported [65]. Endocytosis of extracellular proteins contributes to alpha granule content [40]. Rab31 appears to play an important role in this process as endosomal trafficking is impaired and alpha granule proteins like VWF accumulate in early endosomes of megakaryocytes lacking Rab31 [66]. Furthermore, endocytosed fibrinogen was detected in Rab5 positive granules equivalent to early endosomes, followed by Rab7 positive late endosomes, and finally P-selectin positive alpha granules of megakaryocytes [67]. Rab5 has also been shown to regulate endocytosis and trafficking of the GPIb receptor in megakaryocytes which was linked to proplatelet formation [68]. Alpha granule release might involve Rab4 based on studies using permeabilized platelets [69]. Further characterization of the localization and function of Rab proteins and their GEFs and GAPs might help in clarifying open questions regarding alpha granule subtypes with different release kinetics [70].

Rab regulated endocytotic pathways contribute to the recycling of plasma membrane proteins. Fibrinogen labelled integrin αIIbβ3, passes through a Rab4-positive early endosome compartment followed by colocalization with Rab11-positive structures which are considered recycling endosomes [42]. Similarly, the P2Y_12_ ADP receptor was shown to be recycled via a Rab5/Rab11 pathway in transfected cells [71]. Recycling pathways have also been shown to play a role in virus entry into platelets. HIV virions sequentially pass through Rab4 and Rab7 compartments in platelets finally accumulating in microtubule-associated protein 1A/1B-light chain 3 (LC3) positive compartments similar to those found in phagocytes [41]. Related pathways may be used for the internalization of influenza and/or SARS-COV-2 viruses leading to platelet activation [72,73]. Rab11A and B are actually among the most highly expressed Rabs in platelets (Table 1) pointing towards a prominent role for recycling pathways in platelet function. Furthermore, Rab11 might be cooperating closely with Arf6 in controlling integrin traffic as has been shown for neurons [74].

### RabGAPs

A PubMed search using the terms ‘RabGAP’ and ‘platelets’ did not detect any platelet or megakaryocyte studies at this time. However, one might speculate about potential functions of the top three RabGAPs expressed in platelets based on data from other cells (Figure 2B). TBC1D13, the most highly expressed RabGAP, has been shown to interact with Rab10, to have GAP activity towards Rab35, and to regulate trafficking of membrane proteins towards the plasma membrane [75]. Both, Rab10 and Rab35, are also expressed in platelets (Table 1). TBC1D15, the second highest RabGAP, serves as GAP for Rab7 and regulates mitochondrial fission and regeneration of lysosomes [76,77]. And RabGAP1, the third most highly expressed RabGAP, is involved in recycling of integrins to the plasma membrane [78] pointing to potential cross-talk between RabGAP1 and some of the ArfGAPs like ASAP1 and GIT1 mentioned above.

Taken together, Rabs have been assigned specific roles in the biogenesis and release of dense granules as well as in endocytosis. Further analysis of the localization and function of Rab proteins and their GAPs is required to better understand alpha granules and their possible heterogeneity, as well as membrane recycling pathways, and might help in deciphering the exact roles of the multiple membrane bound compartments found in platelets including the canalicular systems.

## Ras/Rap proteins and Ras/RapGAPs in platelets

### Ras/Rap proteins

The Ras family proteins Rap1A and Rap1B are the most highly expressed small G proteins in platelets. With estimated copy numbers of 120,000 and 150,000 each per platelet [18], they outnumber other G-proteins by about 10-fold (Table 1). The key role of Rap1 proteins appears to be regulation of integrin α_IIb_β_3_ activation and platelet aggregation [79]. Rap1-GTP interacts with talin at the plasma membrane triggering a major conformational change in integrin α_IIb_β_3_ enabling fibrinogen binding and cross-linking of platelets. Platelet activation correlates with increased levels of Rap1-GTP whereas cyclic nucleotide mediated platelet inhibition reduces Rap1-GTP [80,81]. Reduced Rap1 activation for example due to mutation of the gene for CalDAG-GEFI, a Rap1 GEF, causes bleeding in humans and mice [82–86] which has been confirmed in Rap1A and Rap1B knockout mouse models [87–89]. Additional roles for the Rap1A isoform beyond integrin regulation include cross-activation of the Rho family G protein Rac1 as well as a regulation of alpha granule release in response to GPVI activation through unknown mechanisms [88]. Other Ras/Rap1 proteins expressed at higher levels in platelets include Rap2A, B, and C, RalA and B (mentioned above), and the prototypic Ras proteins KRas and NRas. The functions of these G proteins in platelets are not clear. However, RRas2 (TC21), a Ras family member detected in platelets, regulates Rap1B and integrin α_IIb_β_3_ downstream of the GPVI receptor [90].

### Ras/RapGAPs

RASA3 (GAP1-IP4BP) has been confirmed as GAP of Rap1 in megakaryocytes and platelets (Figures 2C and 3A). Complete deletion of RASA3 in megakaryocytes is embryonically lethal due to bleeding and defects in the development of the lymphatic system [91]. Megakaryocytes expressing catalytically inactive or reduced levels of RASA3 exhibit altered adhesive properties, reduced motility and structural alterations [92–94]. As expected, integrin αIIbβ3 was found to be constitutively activated in these cells which correlated with increased Rap1-GTP levels [94]. Basal and ADP stimulated levels of Rap1GTP and active αIIbβ3 were also found to be elevated in mature mouse platelets expressing reduced levels of H794L mutant RASA3 [91]. In particular, RASA3 inhibition was suggested to play a specific role in platelet activation downstream of the P2Y_12_ Gi coupled ADP receptor as P2Y_12_ and PI3K inhibitors lost their in efficiency in RASA3 mutant platelets. Interestingly, RASA3 was also detected in a proteomics approach aimed at identifying phosphatidylinositol 3,4,5-tris-phosphate (PI(3,4,5)P_3_)-binding proteins in platelets [95]. ADP binding to the P2Y_12_ receptor increased the association of RASA3 with the plasma membrane resulting in reduced GAP activity. These studies led to the proposal that the P2Y_12_ receptor might regulate RASA3 through Gi mediated activation of PI3K leading to the production of PI(3,4,5)P_3_ followed by recruitment and inactivation of RASA3 at the plasma membrane (Figure 3A). In this way, RASA3 mediated turnover of Rap1-GTP could be prevented during ADP-induced platelet activation [95]. RASA3 has been described as dual specificity GAP that is able to regulate both, Rap1 and Ras [96] and PI(3,4,5)P_3_ binding could potentially affect the specificity of RASA3 towards Rap1 or Ras. The N-terminal C2 and C-terminal PH domains of RASA3 involved in membrane binding are required for GAP activity towards Rap1, whereas the isolated GAP domain might be sufficient for activity towards Ras [28,97]. Thus, one might speculate that membrane recruitment could shift the activity of RASA3 away from Rap1 towards Ras. However, RASA3 has also been shown to bind to both, PI(4,5)P_2_ as well as PI(3,4,5)P_3_ via its PH domain [98]. In this study, constitutive association of transfected RASA3 with the plasma membrane was observed and PI3K inhibitors had no effect on plasma membrane binding.

Recently, a PI3K independent pathway of Rap1 regulation downstream of P2Y_12_ has been proposed [99], which could potentially involve Rap1GAP2, another highly expressed GAP of Rap1 in human platelets (Figure 2C) [100]. Mouse platelets contain only low levels of Rap1GAP2, whereas humans express Rap1GAP2 at the highest level in platelets compared with other tissues [101] (Table 2). Rap1GAP2 is regulated by activating and inhibitory signalling pathways (Figure 3B). During platelet activation Rap1GAP2 is phosphorylated on serine 9 leading to binding of the adapter protein 14-3-3. 14-3-3 binding correlates with reduced Rap1GAP2 function [102], however, the kinase mediating serine 9 phosphorylation has not yet been identified. In contrast, inhibitory cyclic nucleotide pathways phosphorylate Rap1GAP2 on serine 7 leading to detachment of 14-3-3 (Figure 3B). Thus platelet activators appear to reduce Rap1GAP2 activity to retain high Rap1-GTP levels required for aggregation, whereas platelet inhibitors like the endothelium-dependent cyclic nucleotide pathways reverse this effect. In addition, a TKxT motif in the C-terminal part of Rap1GAP2 (amino acids 524-527) was shown to mediate binding of Rap1GAP2 to synaptotagmin-like protein 1 (Slp1), a protein regulating dense granule release (Figure 3B) [33]. Thus, Rap1GAP2 might link two important platelet responses, aggregation via Rap1 regulation as well as dense granule release via Slp1 binding.

The RasGAP domain of IQGAPs is lacking GAP activity (Figure 2C). Instead, IQGAPs are acting primarily as scaffolds linking receptors to intracellular signalling networks [103]. IQGAPs bind to Rho family G proteins like Racs, RhoG and Cdc42 stabilizing their active GTP-bound forms as well as to Arf6, Rap1 and GDP-bound Rab27a [104,105]. Studies of IQGAP1 deficient platelets indicate that IQGAP1 inhibits intracellular calcium elevation in response to thrombin exposure. IQGAP1 also inhibits alpha granule release and attenuates the development of a procoagulant plasma membrane [106]. IQGAP2, a particularly highly expressed protein in platelets, interacts with the actin-binding protein Arp3 in thrombin treated platelets [107].

In summary, Rap1 has attracted a lot of attention as highly expressed G protein and integrin α_IIb_β_3_ regulator leading to the discovery of RASA3 and Rap1GAP2 as major RapGAPs. However, many open questions remain regarding the regulation of these GAPs and further studies are required to identify the specific roles of the other Ras proteins like Ral, Rap2 or NRas.

## Rho proteins and RhoGAPs in platelets

### Rho proteins

Rac1, Rac2, Cdc42, RhoA, RhoC, and RhoG are the most highly expressed Rho family proteins in platelets, and RhoF is strikingly platelet specific [18] (Table 1). These G proteins appear to have multiple roles in controlling platelet signalling, cytoskeletal reorganization, granule release, aggregation, and clot retraction [13]. Rac1 is essential for signalling downstream of GPVI and CLEC2 receptors activation [108], and both, Rac1 and Rac2, control lamellipodia formation and platelet spreading and are required for the stabilization of platelet aggregates [109]. Cytoplasmic FMR1 Interacting Protein 1 (CYFIP1) has been identified as Rac1 effector mediating lamellipodia formation in platelets [110]. Cdc42 has inhibitory roles in alpha and dense granule release correlating with increased aggregation of knockout platelets [111] although opposing data have been obtained in a different Cdc42 knockout model [112]. RhoA plays a role in shape change, alpha and dense granule release, and clot retraction downstream of Gα_13_-coupled thrombin and TXA_2_ receptors of mature platelets [113]. Target proteins mediating the effects of RhoA-GTP include the Rho-associated protein kinases 1 and 2 (ROCK1/2); however, deletion of these proteins only partly copied the RhoA knockout phenotype [114,115]. For example, loss of ROCK2 lead to reduced Thr853 phosphorylation of myosin phosphatase target 1 and to reduced platelet adhesion [115]. Deletion of other RhoA effectors like Protein diaphanous homolog 1 (mDia1) and FH1/FH2 domain-containing protein 1 (Fhod1) did not result in platelet defects suggesting multiple compensatory Rho effector pathways [116]. A normal phenotype was also described for RhoF mouse knockout platelets again indicating compensatory mechanisms [117]; however, RhoF is expressed only at very low levels in mouse compared to human platelets [101]. On the other hand, RhoG is present at high levels in mouse platelets and RhoG knockout leads to defect in alpha and dense granule release specifically in response to GPVI activation [118,119]. Platelet activation is often associated with the formation of the GTP-bound versions of Rho family proteins, whereas endothelial platelet inhibitors tend to keep these proteins inactive [10].

### RhoGAPs

Thirty-five RhoGAPs have been detected in platelets [18] (Table 2 and Figure 2D), and the G protein/GAP ratio appears to be particularly low for this group of proteins suggesting that Rho proteins might be regulated by more than one GAP per G protein. The specificities of all RhoGAPs towards RhoA, Rac1, and Cdc42 have recently been studied by Müller et al. using a cell-based assay and these data are included in Table 2 [26]. However, as only RhoA, Rac1, and Cdc42 were tested, activities towards other Rho family G proteins remain unknown. Using affinity purification of overexpressed GAPs and GEFs coupled to mass spectrometry Müller et al. also provided evidence for interactions among GAP family members as well as between GAPs and GEFs. In addition, information on spatial distribution was obtained, indicating that many RhoGAPs associate with the actin cytoskeleton, at least in transfected cells.

RhoGAP1 (ARHGAP1) and 18 (ARHGAP18) are the most highly expressed RhoGAPs in platelets (Table 2 and Figure 2D). RhoGAP1 contains a CRAL-TRIO domain (BNIP-2 and Cdc42GAP Homology domain (BCH) subclass) that can bind RhoA with its prenylation moiety resulting in GAP activation [120]. Interestingly, an amino acid change at the start of the catalytic GAP domain of RhoGAP1 (L263F) was identified as putative causal variant in a patient with a primary platelet secretion defect [121]. RhoGAP18 is known to contribute to localized RhoA regulation in distinct cellular compartments [122–124]; however, platelet functions of RhoGAP18 have not yet been described. RhoGAP6 (ARHGAP6), another highly expressed RhoGAP, has been shown to bind to δ-COP, a component of the COPI coat, in platelets (Figure 3C). COPI is usually thought to be involved in vesicle transport between the Golgi and the ER and COPI function requires Arf1 and ArfGAP1-3 [37,125]. But only ArfGAP2 appears to be expressed in platelets at detectable levels (Figure 2A). δ-COP binding involves three tryptophan motifs (LIG_deltaCOP1_diTrp_1 according to the ELM resource [126]) in the C-terminal part of RhoGAP6 (amino acids 947-958). This tryptophan motif has previously been characterized only in ArfGAP1 [27]. The δ-COP interaction is inhibited by 14-3-3 binding to phosphorylated serine 37, and RhoGAP6 stimulates vesicle transport and protein secretion in transfected cells which depends on COPI binding but not on GAP activity (Figure 3C). Taken together RhoGAP6 might link RhoA regulation with protein transport processes in platelets.

Other RhoGAPs that have been studied in platelets include oligophrenin 1 (OPHN1), RhoGAP21 (ARHGAP21), Oculocerebrorenal syndrome of Lowe 1 and Inositol 5-phosphatase (OCRL), RhoGAP17 (ARHGAP17), and Myo9B (Table 2). Oligophrenin 1 inhibits lamellipodia formation and adhesion, alpha and dense granule release and thrombus formation under low shear rates, whereas platelet aggregation does not appear to be regulated according to a mouse study [127]. In this study evidence was provided for GAP activity of oligophrenin towards RhoA, Rac1, as well as Cdc42; however, other Rho family proteins have not been tested. Similarly, investigation of RhoGAP21 in mouse platelets revealed a role for this GAP in attenuating alpha granule release, platelet aggregation, and thrombus formation which was linked to inactivation of RhoA and Cdc42 [128]. In the GAP domain of OCRL, the catalytic arginine is replaced by a glutamine and no GAP activity can be detected, nevertheless OCLR has been shown to bind to Rac1-GTP [129]. OCRL also contains a central inositol 5-phosphatase phosphatase domain processing PIP_2_ into phosphatidylinositol 4-phosphate (PI_4_P). Defects in OCRL cause a rare inherited disorder which is associated with a bleeding phenotype. A recent study of platelets from these patients showed reduced adhesion to collagen surfaces, enhanced filopodia and reduced lamellipodia formation on fibrinogen, which correlated with reduced Rac1-GTP and enhanced RhoA-GTP levels but reduced myosin light chain phosphorylation, clot retraction, and thrombus formation [130]. Similar findings were obtained by pharmacological inhibition of OCRL’s phosphatase activity including enhanced filopodia and actin nodule formation, reduced spreading and reduced myosin light chain phosphorylation [131]. Taken together, OCRL might regulate actin dynamics by Rac1 binding as well as by lowering PIP_2_ levels in the plasma membrane with follow-on effects on PIP_2_-binding proteins. RhoGAP17 is another GAP with activities towards Rac1 and RhoA found in platelets. RhoGAP17 has been identified as a target of various signalling pathways. PKA and PKG phosphorylate RhoGAP17 on serine 702 leading to detachment of the Cdc42-interacting protein 4 (CIP4) from RhoGAP17 [51]. CIP4 is an effector of Rac1-GTP and Cdc42-GTP involved in proplatelet formation [132,133]. Loss of CIP4 binding to RhoGAP17 correlates with enhanced inhibition of cell migration possibly through increased GAP activity leading to reduced Rac1-GTP levels [51]. Thus, PKA/G-mediated detachment of CIP4 might activate RhoGAP17 and terminate Rac1 signalling towards CIP4 (Figure 3D). Interestingly, RhoGAP17 can also be activated by tyrosine phosphorylation (Y124 and Y314) through Src family kinases during platelet activation [134] suggesting that both, platelet activation and inhibition pathways, might lead to RhoGAP17 activation. These phosphorylations could potentially have additive effects on GAP activity levels; however, cross-regulation of RhoGAP17 phosphorylation sites has not been investigated. PKA/G also phosphorylate the RhoGAP and myosin motor protein Myo9B which moves along actin filaments and has been implied in lamellipodia formation in various cells [135,136]. Phosphorylation of Myo9B on serine 1354 enhanced the GAP activity of Myo9B towards RhoA [137] possibly contributing to a modulation of local RhoA activity around actin filaments [138].

In summary, Rho family G proteins are appearing to be regulated by a complex set of RhoGAPs integrating numerous additional domains and functions. Specific combinations of Rhos and GAPs/GEFs might be involved in controlling distinct cellular processes beyond the classical roles of RhoA, Cdc42, and Rac1 in stress fibre, filopodia and lamellipodia formation.

## Heterotrimeric G proteins and RGSs in platelets

### Heterotrimeric G proteins

Heterotrimeric (αβγ) G proteins transduce signals from ligand binding GPCRs to downstream targets at the plasma membrane [139]. Platelets express all 3 Gα_i_ isoforms (Gα_i1-3_) and Gα_q_ at highest levels [18] (Table 1). Gα_13_, Gα_z_ (another G_i_ family member), and Gα_s_ are less prominent. The most highly expressed corresponding beta and gamma subunits are β1, β4, β2, β5, and γ11, γ5, γ10 (in descending order of expression level). Activated GPCRs act as GEFs and GTP-bound α subunits dissociate from βγ subunits to interact with downstream effector targets. Activation of the P2Y_12_ receptor by ADP induces Gα_i2_- and Gα_z_-GTP formation which inhibits AC leading to reduced cAMP synthesis and facilitating platelet activation and aggregation [140–142]. Overexpression of a GAP resistant Gα_i2_ mutant led to reduced cAMP levels, and increased aggregation in response to GPCR agonists confirming the inhibitory role of Gα_i_ in platelets [143]. The P2Y_1_ ADP receptor as well as thrombin and TXA_2_ receptors activate Gα_q_ (Figure 1) [144,145] leading to stimulation of PLCβ followed by the release of Ca^2+^ ions from intracellular stores and platelet activation [146,147]. The PAR1 thrombin receptor and the TP receptor are also linked to Gα_13_ which activates RhoA [148] probably through direct interaction with a RhoGEF like RhoGEF1 (p115RhoGEF) [149]. Information on the role of G_s_, linked to the prostacyclin receptor (IP receptor), has been obtained using G_s_-deficient platelets from patients with pseudohypoparathyroidism types Ia [150,151]. In these platelets reduced G_s_ expression results in attenuated responses to IP receptor activation including reduced AC stimulation, followed by reduced cAMP/PKA activation and reduced inhibition of platelet aggregation. G_s_ overexpression has been associated with an increased bleeding risk [152]. Only few studies have addressed roles of Gβγ subunits of heterotrimeric G proteins in platelets. Gβ_1_ was shown to interact with the catalytic subunit of protein phosphatase 1 and with PLCβ3 [153], and a small molecule inhibitor of Gβγ inhibits platelet aggregation and granule release [154]. Gβγ subunits contribute to PI3K activation downstream of G_i_-coupled receptors [155,156] and to AC activation downstream of G_s_-coupled receptors [157]. Multiple combinations of α, β and γ subunits are expected to exist leading to many possible functions [158].

### RGSs

GAP roles are provided by RGS proteins some of which have been studied in platelets (Table 2 and Figure 2E). Only 20 (RGS1-20) of the 35 proteins containing RGS or RGS-homology (RH) domains are considered canonical RGS proteins based on structural and functional (GAP activity) similarities [25,159]. RGS18 is the most highly expressed and platelet-specific RGS (Table 2) and exhibits selectivity towards Gα_q_ and Gα_i_ (Figure 3E) [159]. Knockout mouse studies revealed a role for RGS18 in megakaryocyte development and platelet production [160,161]. Mature platelets lacking RGS18 are hyperactive exhibiting increased basal as well as thrombin and TXA_2_ stimulated alpha and dense granule release, integrin activation and aggregation. RGS18 interacts with Gα_q_ and Gα_i_ in human platelets, as well as with the adapter proteins spinophilin (neurabin-2, PPP1R9B), and 14-3-3 [162–165]. A regulatory cycle was proposed for RGS18 leading to RGS inhibition during platelet activation (associated with 14-3-3 binding to phosphorylated serines 49 and 218 and loss of spinophilin binding) and activation during platelet inhibition (associated with cyclic nucleotide mediated loss of 14-3-3 due to S216 phosphorylation) (Figure 3E) [10] PKA-mediated phosphorylation of serine 94 on spinophilin contributes to dissolution of the RGS18/14-3-3/spinophilin complex [165]. Recruitment of the serine/threonine phosphatase PP1 to spinophilin is regulated by the tyrosine phosphatase SHP-1 [166] and might play a role in dephosphorylating the regulatory sites on RGS18 [163]. A recent study has revealed further complexity of heterotrimeric G protein regulation as Gα_q_ was shown to interact with either RGS10 or RGS18 or with PLCβ [167]. RGS10 is another via Gα_q_/Gα_i_-specific abundantly expressed RGS in platelets which has been shown to negatively regulate release of calcium ions from intracellular stores, alpha granule release, aggregation, and thrombus formation downstream of thrombin, TXA_2_, and ADP receptors in mice and to be regulated by spinophilin and 14-3-3 binding similar to RGS18 [168–170]. RGS16 has been described as a platelet regulator [171]; however, proteomics have provided little evidence for RGS16 expression in platelets (Table 2).

In addition to the classical RGS proteins a few related proteins containing RH domains are expressed in platelets. The G protein–coupled receptor kinases (GRK) appear to have some GAP activity towards Gα, but many of these have numerous ways of regulating receptor functions [25]. GRK2 has been shown to specifically terminate P2Y_12_ and P2Y_1_ coupled G_q_ and possibly G_i_ signalling in platelets [172]. Of note, in this study, GRK2 was seen to bind to Gβ_q_ but not the Gα_q_ or Gα_i_ subunits. In contrast, GRK6 regulates PAR1 receptors. GRK6 has been shown to bind to the PAR1 receptor in thrombin treated platelets which was linked to increased receptor phosphorylation but GRK6 might also regulate PAR4, at least in mice [173]. GRK5 also regulates PAR1 and PAR4 mediated platelet activation in a mouse knockout model where lack of GRK5 led to increased thrombin-induced aggregation. The common intronic GRK5 variant rs1088643 linked to reduced transcript numbers is associated with increased thrombin-induced platelet reactivity in humans [174,175]. As GRK5 was shown to bind to the PAR1 receptor GRK5 might de-activate PAR signalling through phosphorylation and β-arrestin mediated receptor internalization.

Taken together, RGS proteins are emerging as essential regulators of GPCR function in platelets. RGS18 has been shown to regulate transmission of G_q_ and G_i_ mediated thrombin and TXA_2_ receptor signalling in many studies. Further analyses are required to clarify GPCR specificities and regulatory complexes forming around RGSs.

## Platelet G proteins and GAPs as therapeutic targets

Platelets play a major role in arterial and venous thrombotic disease. However, current antiplatelet therapies are targeting only a restricted set of molecules like the P2Y_12_ ADP receptor, cyclooxygenase-1 required for TXA_2_ synthesis, or the cAMP degrading phosphodiesterase type 3 [176,177]. Furthermore, the major drawback of any anti-haemostatic approach, an increased bleeding risk, has not been overcome. G proteins and their regulators might represent a new class of targets for antiplatelet therapy.

The approval of sotorasib in 2021 as first G protein inhibitor targeting K-Ras in cancer patients indicates the feasibility of targeting these types of proteins [178]. Both, GTP- and GDP-bound states of K-Ras can be targeted by small molecules, and drugs are being developed that interfere with effector and/or GEF and GAP binding sites. Highly expressed and platelet specific G proteins like Rap1B or Rab27B could be promising targets for the development of similar drugs for antiplatelet therapy. In principal, it should also be possible to identify small molecules that stimulate GAP functions, for example, by interfering with negative regulatory regions. The platelet specific Rap1GAP RASA3 could potentially be activated by interference with the regulatory PH domain of RASA3 whereas blocking 14-3-3 interaction would activate Rap1GAP2 (Figure 3A,B). Similarly, impeding interactions between RGS18 and spinophilin or 14-3-3 would be expected to lead to platelet inhibition (Figure 3E) and the development of 14-3-3 interaction inhibitors is an area of active research [179]. As GAPs are regulated by complex phosphorylation patters targeting protein kinases might be another way of influencing platelet function. Kinase inhibitors have become a major area of drug development since the discovery and clinical implementation of imatinib as highly effective inhibitor of the tyrosine kinase BCR-ABL in cancer therapy. Around 20 kinase inhibitors are already in clinical use and 20% of the entire kinome of 560 kinases is being targeted in clinical trials [180]. Studies are also underway addressing the potential of PI3K and tyrosine kinase inhibitors for platelet inhibition [176]. Kinases appear to have a major role in regulating GAPs (Table 2), however, further studies are required to identify specific kinase/GAP interactions with therapeutic potential in platelets.

## Conclusion

Platelets contain a unique set of G proteins and G protein regulators which are likely to be adapted to the specialised functions of this cell type. So far only few platelet GAPs have been studied in detail. The multi-domain structure of GAPs might contribute to local control of G proteins and facilitate complex connections between G proteins of different families required for the regulation of receptor function, membrane and vesicle traffic and the cytoskeleton. Platelets represent a unique cell model for signalling research as gene expression changes do not need to be considered. In addition to platelet focused omics studies careful validation of the specificities, control mechanisms, and localization of individual proteins will be essential to build a solid foundation for improved understanding of platelet function, meaningful modelling of signalling networks, and for the identification of new therapeutic targets in vascular disease.

## Abbreviations

AP: adapter protein
cGMP: cyclic guanosine monophosphate
CIP4: Cdc42-interacting protein 4
COPI: coat protein complex I
CYFIP1: cytoplasmic FMR1 interacting protein 1
DAG: 1,2-diacylglycerol
FcRγ: Fc gamma receptor
Fhod1: FH1/FH2 domain-containing protein 1
GAP: GTPase-activating protein
GEF: guanine nucleotide exchange factor
HPS: Hermansky–Pudlak syndrome
IP_3_: inositol 1,4,5-triphosphate
OCRL: Oculocerebrorenal syndrome of Lowe 1 and Inositol 5-phosphatase
PKG: protein kinase G
ROCK1/2: Rho-associated protein kinases 1 and 2
Slp1: synaptotagmin-like protein 1
SNARE: soluble N-ethylmaleimide-sensitive factor attachment protein receptor
